# Outcomes of *Clostridium difficile* Infection in Patients With Idiopathic Pulmonary Fibrosis: Analysis of the Nationwide Inpatient Sample, 2016–2020

**DOI:** 10.1002/jgh3.70416

**Published:** 2026-05-11

**Authors:** Chun Ho Szeto, Gloria Erazo Montalvan, Chung Yin Tai, Edgar Luna Landa, Godfrey Tabowei, Raymond Wadie, Raghavendra Sanivarapu

**Affiliations:** ^1^ Department of Internal Medicine Texas Tech University Health Science Center Permian Basin Odessa Texas USA; ^2^ Department of Orthopaedics and Traumatology The University of Hong Kong Pok Fu Lam Hong Kong; ^3^ Library, The Education University of Hong Kong Tai Po Hong Kong; ^4^ Department of Internal Medicine, Cayuga Medical Center Ithaca New York USA

**Keywords:** *Clostridium difficile*, idiopathic pulmonary fibrosis, interstitial lung diseases, propensity score

## Abstract

**Background:**

Idiopathic pulmonary fibrosis (IPF) is a progressive lung disease with poor prognosis. Patients with IPF are frequently exposed to risk factors for *Clostridium difficile* infection (CDI), such as advanced age, antibiotic use, and gastric acid suppression. However, the outcomes of CDI in hospitalized IPF patients remain unclear.

**Methods:**

We conducted a retrospective cohort study using the National Inpatient Sample (2016–2020) to compare outcomes among hospitalized CDI adults with or without IPF as a pre‐existing comorbidity. Hospitalizations were identified using ICD‐10 codes. Propensity score matching (1:4) was performed to balance demographics and comorbidities. The primary outcome was all‐cause in‐hospital mortality; secondary outcomes included length of stay, hospitalization costs, mechanical ventilation, vasopressor use, and major adverse events.

**Results:**

Of 1 450 860 CDI hospitalizations, 1600 (0.11%) had IPF. After matching, IPF was independently associated with higher in‐hospital mortality (aOR 1.85, 95% CI 1.22–2.76), longer hospital stays, and increased hospitalization costs. IPF patients were more likely to require mechanical ventilation (aOR 1.79, 95% CI 1.27–2.52). However, rates of fulminant CDI complications (severe sepsis, toxic megacolon, ileus, colectomy, ileostomy) were not significantly increased in the IPF cohort after matching.

**Conclusions:**

Hospitalized CDI patients with IPF experience significantly higher mortality and resource utilization compared to those without IPF. The absence of increased fulminant CDI complications suggests that CDI may act as a marker of underlying disease severity in IPF rather than a direct cause of death. These findings highlight the need for risk stratification in CDI patients with chronic lung diseases.

## 1 Introduction

Idiopathic pulmonary fibrosis (IPF) is the most common form of idiopathic interstitial pneumonias, characterized by progressive fibrosis of lung parenchyma and declining lung function. The estimated incidence of IPF ranged from 0.09 to 1.30 per 10 000 population globally, and 0.75 to 0.93 per 10 000 in North America [1]. IPF generally carries a poor prognosis, with a median survival time of 2–3 years following diagnosis [2]. Current treatment guidelines for IPF recommend a personalized approach involving antifibrotic agents, oxygen supplementation, and management of comorbidities and symptoms [3]. Lung transplantation remains the definitive treatment for IPF [3]. In the context of acute IPF exacerbation, most patients received antibiotics in addition to corticosteroids, despite the lack of strong evidence supporting this practice [4].

*Clostridium difficile* (C. diff) is the most common pathogen responsible for healthcare‐associated infections in the United States, accounting for 15% of all such infections [5]. Identified risk factors of C. diff infection (CDI) include advanced age, multiple comorbidities, antibiotic use, gastric acid suppression, immunosuppression, residence at long‐term institutions and acute‐care hospitals, all of which are common among IPF patients [6].

While shared risk factors suggest IPF patients may develop CDI during hospitalization, it remains unclear whether pre‐existing IPF modifies the severity and outcomes of CDI when it occurs. Understanding the prognostic impact of pulmonary comorbidities in CDI patients is essential for risk stratification and resource allocation. Therefore, we conducted an exploratory comorbidity analysis to compare clinical outcomes among hospitalized CDI patients stratified by the presence or absence of IPF.

## 2 Materials and Methods

### 2.1 Data Source

This was a retrospective cohort study using data from the National Inpatient Sample (NIS) between January 1, 2016 and December 31, 2020. The NIS is part of the Healthcare Cost and Utilization Project (HCUP), sponsored by the Agency for Healthcare Research and Quality [7]. The NIS is the largest, publicly available, all‐payer, inpatient database in the United States, excluding rehabilitation and long‐term acute care hospitalization facilities. The data represent stratified samples of 20% of all discharge records in the United States, covering nearly 95% of the United States population. Institutional review board approval and informed consent were waived for this study in accordance with the Declaration of Helsinki, as patient confidentiality was protected through de‐identification.

**FIGURE 1:**
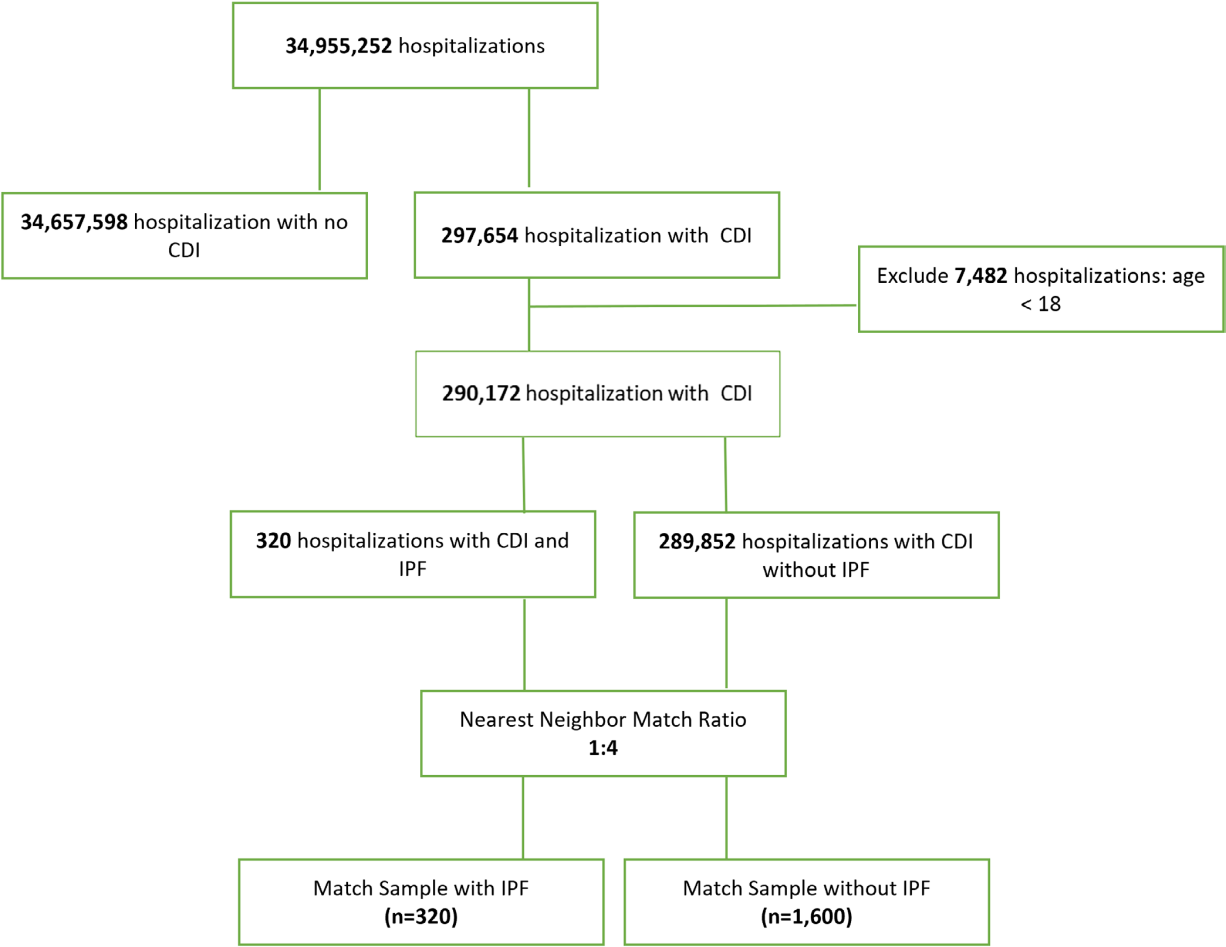
Cohort derivation flow chart.

We included hospitalizations from January 1, 2016 to December 31, 2020, using data from the NIH‐HCUP database, which applies the International Classification of Diseases, 10th Revision (ICD‐10) codes [8]. ICD‐10 codes were used to identify hospitalizations primarily for CDI (A047, A0471, A0472). Within this cohort, patients with a concomitant diagnosis of IPF (J84112) were identified for comparison against CDI patients without IPF. Patients under 18 years of age were excluded, as IPF is an adult‐onset interstitial lung disease, with pediatric cases being rare and pathobiologically distinct from adult IPF. A flow chart illustrating the selection process is shown in Figure 1.

The primary outcome was all‐cause‐in‐hospital mortality among patients hospitalized with CDI. Secondary outcomes included costs of hospitalizations, length of stay, and in‐hospital major adverse events, such as mechanical ventilation, vasopressor use, severe sepsis, and septic shock, ileostomy, and colectomy. The ICD‐10 codes used to identify these outcomes are provided in Table S1.

### 2.2 Statistical Analysis

Weighted estimates were generated by applying survey trend weights to account for data stratification as recommended by the HCUP. Continuous variables were presented as means and standard deviations, and the categorical variables were presented as percentages. We compared differences in baseline characteristics between the two study groups, including age, sex, race, payer, and comorbidities, using an independent sample's *t*‐test for continuous variables and chi‐square and Fisher's exact tests for categorical variables. Hospitalizations with missing data on age, sex, and in‐hospital mortality were imputed by the *k*‐nearest neighbors method [9].

To decrease the differences in the baseline characteristics and increase comparability between IPF and non‐IPF groups, we performed propensity score matching (PSM) analysis. The PSM methodology balances interference factors between subgroups in observational studies. First, we derived a propensity score using multivariable logistic regression that models the IPF as the dependent variable and all the baseline characteristics. Second, matching was performed using a nearest‐neighbor 1:4, applying a caliper width of 0.20 standard deviation of the logit of the propensity score to prevent poor matches. Covariate balance was evaluated with absolute standardized mean differences (SMDs) before and after matching, considering an SMD < 0.10 as adequate balance. Third, within the matched cohort, outcome analyses followed a distribution‐conscious approach. Binary end‐points—including in‐hospital death, mechanical ventilation, vasopressor use, severe sepsis/septic shock, acute kidney injury, ileus, colonic perforation, colectomy and ileostomy/colostomy—were modeled with Firth‐penalized logistic regression, which provides finite, bias‐reduced odds‐ratio (OR) estimates in the presence of rare events or separation. Length of stay, treated as an over‐dispersed count, was analyzed with a negative‐binomial generalized linear model, and exponentiated coefficients were interpreted as incidence‐rate ratios (RR). Total hospital charges were analyzed with a Gamma log‐link model and reported as multiplicative cost ratios (RR). For clinical interpretability, adjusted marginal means for length of stay and charges were additionally estimated, and the absolute differences (IPF minus non‐IPF) were presented alongside the relative effects. Parallel unadjusted models (IPF as the sole predictor) were fitted to allow direct comparison of crude and covariate‐adjusted estimates. Statistical significance was declared at two‐sided *p* < 0.05. Data manipulation and statistical analyses were performed using R statistical software (version 3.5; R Foundation for Statistical Computing). PSM was performed using the Matchit package in R statistical software.

## 3 Results

### 3.1 Baseline Characteristics

From 2016 to 2020, 1 488 270 weighted records with CDI were identified. Of these, 1 450 860 hospitalizations met inclusion criteria. Of these hospitalizations, 1600 (0.11%) carried a diagnosis of IPF, and the rest 1 449 260 (99.89%) were included in the non‐IPF cohort. The mean ages of the patients in the IPF and non‐IPF cohorts were 71.99 years and 66.15 years, respectively. Patients in the IPF group were more likely to be White and male. Comorbidities such as congestive heart failure, hypertension, chronic pulmonary diseases, and gastroesophageal reflux disease (GERD) were more common in IPF patients, whereas liver disease, chronic kidney disease, obesity, and diabetes were more prevalent in the non‐IPF group (all *p* < 0.05). Baseline demographic characteristics and comorbid conditions are described in Table 1.

**TABLE 1:** Comparison of clinical characteristics in patients with CDI with and without IPF before and after propensity score matching.

Before propensity matching						After propensity matching				
Variable	IPF cohort (*n* = 1600)		Non‐IPF cohort (*n* = 1 449 260)			IPF cohort (*n* = 1600)		Non‐IPF cohort (*n* = 6400)		
	Number	Percent (%)	Number	Percent (%)	*p*	Number	Percent (%)	Number	Percent (%)	*p*
Demographics										
Age (years)	71.99 ± 11.57		66.15 ± 16.72		< 0.001	71.99 ± 11.58		72.31 ± 12.28		0.385
Male	815	50.9	618 900	42.7		815	50.9	3265	51.0	
Female	785	49.1	830 360	57.3		785	49.1	3135	49.0	
Race/ethnicity					< 0.001					0.391
White	1200	75.0	1 068 665	73.7		1200	75.0	5045	78.8	
Black	135	8.4	189 975	13.1		135	8.4	490	7.7	
Hispanic	140	8.8	119 490	8.2		140	8.8	550	8.6	
Asian or Pacific Islander	65	4.1	27 160	1.9		65	4.1	175	2.7	
Native American	5	0.3	10 395	0.7		5	0.3	5	0.1	
Other	55	3.4	33 575	2.3		55	3.4	135	2.1	
Hospital region					0.05					0.684
Northeast	330	20.6	271 930	18.8		330	20.6	1360	21.3	
Midwest	451	25.9	355 865	24.6		415	25.9	1505	23.5	
South	575	35.9	541 195	37.3		575	35.9	2250	35.2	
West	280	17.5	280 270	19.3		280	17.5	1285	20.1	
Location/teaching status of hospital					< 0.001					0.839
Rural	115	7.2	127 015	8.8		115	7.2	420	6.6	
Urban nonteaching	230	14.4	315 595	21.8		230	14.4	865	13.5	
Urban teaching	1255	78.4	1 006 650	69.5		1255	78.4	5115	79.9	
Hospital bedsize					< 0.001					0.489
Small	270	16.9	286 640	19.8		270	16.9	1215	19.0	
Medium	360	22.5	408 165	28.2		360	22.5	1270	19.8	
Large	970	60.6	754 455	52.1		970	60.6	3915	61.2	
Health insuranced < 0.001										
Medicare	1245	77.8	949 380	65.5		1245	77.8	5220	81.6	
Medicaid	45	2.8	176 500	12.2		45	2.8	90	1.4	
Private insurance	270	16.9	260 640	18.0		270	16.9	940	14.7	
Self‐Pay	20	1.2	31 050	2.1		20	1.2	50	0.8	
No charge	0	0.0	2775	0.2		0	0.0	0	0.0	
Other	20	1.2	28 915	2.0		20	1.2	100	1.6	
Hospital disposition < 0.001										
Home or self‐care	355	22.0	566 035	39.1		355	22.0	1940	30.3	
Short‐term hospital for inpatient care	35	2.2	35 360	2.4		35	2.2	120	1.9	
Another facility	575	35.9	470 380	32.5		575	35.9	2385	37.3	
Home with home health service	435	27.2	273 325	18.9		435	27.2	1415	22.1	
Left against medical advice or discontinued care	5	0.3	13 725	0.9		5	0.3	45	0.7	
Expired	195	12.2	90 145	6.2		195	12.2	495	7.7	
Comorbidities										
Congestive heart failure	680	42.5	393 025	27.1	< 0.001	680	42.5	2790	43.6	0.731
Hypertension	1150	71.9	998 315	68.9	0.011	1150	71.9	4640	72.5	0.823
Chronic pulmonary disease	785	49.1	381 675	26.3	< 0.001	785	49.1	3025	47.3	0.570
GERD	585	36.6	347 120	24	< 0.001	585	36.6	1525	23.8	< 0.001
Liver disease	125	8.4	145 740	10.1	0.035	125	8.4	515	8	0.817
Nicotine dependence	575	35.9	491 410	33.9	0.091	575	35.9	2385	37.3	0.669
Chronic kidney disease	440	27.5	459 185	31.7	< 0.001	440	27.5	1865	29.1	0.562
Obesity	145	9.1	212 115	14.6	< 0.001	145	9.1	520	8.1	0.595
Diabetes mellitus	485	30.3	490 860	33.9	0.003	485	30.3	1980	30.9	0.830

Abbreviations: CDI, *Clostridium difficile* infection; GERD, gastroesophageal reflux disease; IPF, idiopathic pulmonary fibrosis; SD, standard deviations.

The 5‐year prevalence of IPF among patients with CDI was 110 per 100 000 CDI hospitalizations. The yearly prevalence of IPF among CDI hospitalizations was 95.4 per 100 000 in 2016 and 99.2 per 100 000 in 2017, compared with 117.1 in 2018, 142.6 in 2019, and 102.1 in 2020.

In the unmatched analysis, all‐cause mortality in the IPF group was 12.2%, significantly higher than 6.2% in the non‐IPF group (aOR 1.67, 95% CI 1.13–2.46, *p* < 0.05) (Table 2). IPF was also associated with longer length of stay and higher total hospital charges compared with non‐IPF hospitalizations. Mechanical ventilation occurred in 290 (18.1%) IPF hospitalizations, significantly higher than in the non‐IPF group (aOR 1.71, 95% CI 1.23–2.39, *p* < 0.05). No significant association was observed in the rates of severe sepsis and septic shock, ileus, acute kidney injury, colonic perforation, colectomy, and ileostomy in the IPF cohort.

**TABLE 2:** Clinical outcomes in the two cohorts of patients with CDI with and without IPF before and after propensity score matching.

Outcome	IPF cohort (*n* = 1600)	Percentage (%)	Non‐IPF cohort (*n* = 1 449 260)	Percentage (%)	Adjusted OR	95% CI lower	95% CI upper	*p*
Before propensity score matching								
Mortality	195	12.2	90 190	6.20%	1.666	1.127	2.463	0.010
Length of stay (days)	15.23^a^		10.98^a^		1.386	1.247	1.544	< 0.001
Total charges of hospitalization (US dollars)	255627.10^a^		118352.20^a^		2.160	1.743	2.703	< 0.001
Comorbidities								
Mechanical ventilation	290	18.1	140 510	9.7	1.713	1.230	2.385	0.001
Vasopressor use	40	2.5	13 940	1.0	2.085	0.903	4.813	0.085
Severe sepsis or shock	205	12.8	153 215	10.6	1.132	0.784	1.636	0.508
Acute kidney injury	600	37.5	511 545	35.3	0.875	0.680	1.126	0.300
Ileus	90	5.6	48 725	3.4	1.376	0.800	2.367	0.248
Colonic perforation	0.0	0.0	6930	0.5	NA	NA	NA	NA
Colectomy	20	1.2	23 385	1.6	0.795	0.287	2.205	0.660
Ileostomy or colostomy	20	1.2	18 025	1.2	1.720	0.566	5.226	0.339

Outcome	IPF cohort (*n* = 1600)	Percentage (%)	Non‐IPF cohort (*n* = 6400)	Percentage (%)	Adjusted OR	95% CI lower	95% CI upper	*p*
After propensity score matching								
Mortality	195	12.2	495	7.7	1.851	1.245	2.751	0.002
Length of stay (days)	14.36^a^		10.60^a^		1.355	1.219	1.508	< 0.001
Total charges of hospitalization (US dollars)	189308.40^a^		99373.19^a^		1.905	1.548	2.362	< 0.001
Comorbidities								
Mechanical ventilation	290	18.1	735	11.5	1.790	1.277	2.510	0.001
Vasopressor use	40	2.5	80	1.3	2.320	1.096	5.038	0.033
Severe sepsis or shock	205	12.8	740	11.6	1.191	0.821	1.727	0.357
Acute kidney injury	600	37.5	2605	40.7	0.938	0.72	1.222	0.635
Ileus	90	5.6	270	4.2	1.406	0.819	2.415	0.217
Colonic perforation	0.0	0.0	55	0.9	NA	NA	NA	NA
Colectomy	20	1.3	110	1.7	0.764	0.298	1.960	0.575
Ileostomy or colostomy	20	1.3	50	0.8	1.579	0.595	4.196	0.359

Abbreviations: CDI, *Clostridium difficile* infection; CI, confidence interval; IPF, idiopathic pulmonary fibrosis; NA, not applicable; OR, odd ratio; US, United States.

^a^ Means.

One‐to‐four (1:4) matching was performed for age, sex, race, payers, bed numbers, facility type and comorbidities, including congestive heart failure, diabetes mellitus, obesity, hypertension, chronic pulmonary diseases, chronic kidney diseases, GERD, liver diseases and nicotine dependence. The cohorts were well matched after propensity score matching (mean standard difference < 0.1 for all covariates).

After propensity score matching, IPF remained significantly associated with in‐hospital mortality (aOR 1.85, 95% CI 1.25–2.75, *p* < 0.05). Mean length of hospitalization was significantly longer in the IPF cohort (mean difference 3.76 days, *p* < 0.001). Mean hospitalization costs were also significantly higher in the IPF group compared to the non‐IPF cohort ($89 935, *p* < 0.001). IPF patients were more likely to require mechanical ventilation (aOR 1.79, 95% CI 1.28–2.51). After matching, IPF patients showed higher odds of requiring vasopressor use (aOR 2.32, 95% CI 1.07–5.03). However, no significant differences were found in the rates of ileus after matching.

## 4 Discussion

To our knowledge, this is the first study to specifically examine the outcome of CDI among hospitalized IPF patients. Our findings showed that hospitalized individuals with IPF who contracted CDI have considerably poorer outcomes compared to those without IPF, including higher in‐hospital mortality, longer hospital stays, and higher total hospitalization charges. These results are consistent with previous studies reporting high in‐hospital mortality among IPF patients, especially those requiring mechanical ventilation [10, 11].

Interestingly, our study found that IPF was not associated with conditions or procedures indicative of fulminant CDI, such as toxic megacolon, ileus, colectomy, and ileostomy. This suggests that CDI is not directly causing excess mortality among IPF patients through gastrointestinal pathology. The excess mortality stems from respiratory and hemodynamic decompensation related to baseline IPF, represented by the increased mechanical ventilation and vasopressor use in IPF‐CDI patients in our study. Nevertheless, CDI in IPF patients may serve as a marker of greater underlying illness severity, frailty, or prolonged hospitalization complexity.

Antibiotic use is a well‐established risk factor for CDI, with broad‐spectrum antibiotics posing an even greater risk [12]. Therefore, the association between CDI and chronic pulmonary diseases that requires frequent antibiotics challenges has drawn researchers' attention. de Miguel‐Díez et al. revealed that the incidence of CDI was higher in chronic obstructive pulmonary disease (COPD) patients than in those without COPD, and the high mortality rates of CDI patients with COPD can be explained by increasing age, presence of comorbidities, severe CDI, longer length of hospitalization and readmission [13]. In cystic fibrosis, C. diff carriage is common, with half of the adults in a cohort carrying C diff., however often asymptomatic [14]. These associations are hypothesized to be due to constant antibiotics challenge among patients with cystic fibrosis and COPD exacerbation [13, 14]. In the context of IPF exacerbation, despite the lack of benefit of antimicrobial agents, patients are often treated with empiric antibiotics, which can potentially increase the risk of gastrointestinal toxicity [4, 15]. In our study, we observed a rising trend of IPF prevalence among CDI hospitalizations from 2016 to 2019, however, these estimates were descriptive in nature and cannot assess antibiotic prescribing patterns or their temporal relationship to CDI. Future studies incorporating detailed medication data are needed to evaluate whether antibiotic stewardship interventions can reduce CDI risk in IPF patients.

Proton pump inhibitor (PPI) use is another recognized risk factor for CDI, with a positive dose‐ and duration‐dependent relationship [16, 17]. PPIs are frequently prescribed to IPF patients, and guidelines recommend against deprescribing PPI in patients with IPF due to the frequent concurrent diagnosis of GERD [3, 18]. However, the benefits of PPIs for IPF mortality and hospitalization incidence remain inconclusive [19]. Although PPI use is common in IPF patients and prior studies have linked PPIs to increased CDI risk, our dataset does not capture PPI exposure, and therefore we cannot evaluate its role in the outcomes observed. Future research should examine whether PPI stewardship strategies can reduce CDI risk and improve outcomes in IPF.

In a supplementary analysis using ICD‐10 code A04.71, 9.97% in the IPF cohort were coded as recurrent CDI, compared to 8.57% in the non‐IPF cohort (*p* = 0.4260). However, this analysis is limited by the fact that ICD‐10 code A04.71 was only activated in October 2017, resulting in systematic undercounting of recurrent CDI across both cohorts during the first 2 years of the study period (2016–2017) [8]. Given that CDI recurrence is independently associated with increased mortality and healthcare resource utilization in the general population, future patient‐level longitudinal analyses are warranted to accurately characterize the burden and determinants of recurrent CDI in patients with IPF [20].

This study has several limitations. First, as the NIS is an administrative database, it is subject to coding and documentation errors. However, NIS employs internal quality control measures to minimize the effect of those errors [21]. Misdiagnosis of IPF is also a possible source of errors, given the complexity of diagnosis [22]. Second, as a retrospective observational study based on discharge data, our study is susceptible to selection bias and cannot establish temporal precedence or causality. Our analysis serves as an exploratory, hypothesis‐generating study, examining whether IPF presence alters CDI outcomes, not whether IPF is more prone to CDI. Additionally, cost analyses are specific to the United States and may not be generalizable to other regions. Finally, the NIS does not provide detailed information regarding IPF progression (FVC, DLCO) and important confounders, such as the type or course of antibiotics, antifibrotics, or PPI use, limiting our ability to assess the impact of these factors on CDI outcomes.

## 5 Conclusion

In this large, national cohort study, we found that hospitalized patients with IPF who develop CDI experience significantly higher in‐hospital mortality, longer hospital stays, and greater healthcare costs compared to those without IPF. Future studies incorporating detailed medication data are needed to clarify how specific treatments, including antibiotics and PPIs, influence CDI risk in this population and to evaluate whether targeted stewardship strategies can improve clinical outcomes.

## Funding

The authors have nothing to report.

## Conflicts of Interest

The authors declare no conflicts of interest.

## Supporting information


**Table S1:** ICD‐10‐CM and CPT codes used to define study variables.

## Data Availability

The data that support the findings of this study are available from the corresponding author upon reasonable request.
