# Supplementary material for: Outcomes of Clostridium difficile Infection in Patients With Idiopathic Pulmonary Fibrosis: Analysis of the Nationwide Inpatient Sample, 2016–2020
Source: JGH Open. 2026 May 11;10(5):e70416. doi: 10.1002/jgh3.70416 (PMC13161549; doi:10.1002/jgh3.70416)
Supplement: Supplementary file 1 — Table S1: ICD‐10‐CM and CPT codes used to define study variables. [file JGH3-10-e70416-s001.docx]

| **Supplementary Table 1: Diagnosis and ICD-10-CM Codes** | |
| --- | --- |
| **Diagnosis** | **ICD-10-CM Codes** |
| Clostridoides Difficile Infection (CDI) | A0471, A0472, A047 |
| IPF (Idiopathic Pulmonary Fibrosis) | J84112 |
| Mechanical Ventilation | 5A1935Z, 5A1945Z, 5A1955Z |
| Vasopressor Use | 3E043XZ, 3E040XZ |
| Severe Sepsis/Septic Shock | R6521 |
| AKI (Acute Kidney Injury) | N170, N171, N172, N178, N179 |
| Toxic Megacolon | K5931 |
| Ileus | K567 |
| Colonic Perforation | K631 |
| Colectomy | 0D5C0Z3, 0D5C0ZZ, 0D5C4Z3, 0D5C4ZZ, 0D5E0Z3, 0D5E0ZZ, 0D5E4Z3, 0D5E4ZZ, 0D5F0Z3, 0D5F0ZZ, 0D5F4Z3, 0D5F4ZZ, 0D5G0Z3, 0D5G0ZZ, 0D5G4Z3, 0D5G4ZZ, 0D5H0Z3, 0D5H0ZZ, 0D5H4Z3, 0D5H4ZZ, 0D5J0Z3, 0D5J0ZZ, 0D5J4Z3, 0D5J4ZZ, 0D5K0Z3, 0D5K0ZZ, 0D5K4Z3, 0D5K4ZZ, 0D5L0Z3, 0D5L0ZZ, 0D5L4Z3, 0D5L4ZZ, 0D5M0Z3, 0D5M0ZZ, 0D5M4Z3, 0D5M4ZZ, 0D5N0Z3, 0D5N0ZZ, 0D5N4Z3, 0D5N4ZZ, 0DBC0ZZ, 0DBC4ZZ, 0DBE0ZZ, 0DBE4ZZ, 0DBF0ZZ, 0DBF4ZZ, 0DBG0ZZ, 0DBG4ZZ, 0DBGFZZ, 0DBH0ZZ, 0DBH4ZZ, 0DBJ0ZZ, 0DBJ4ZZ, 0DBK0ZZ, 0DBK4ZZ, 0DBL0ZZ, 0DBL4ZZ, 0DBLFZZ, 0DBM0ZZ, 0DBM4ZZ, 0DBMFZZ, 0DBN0ZZ, 0DBN4ZZ, 0DBNFZZ, 0DTC0ZZ, 0DTC4ZZ, 0DTE0ZZ, 0DTE4ZZ, 0DTF0ZZ, 0DTF4ZZ, 0DTG0ZZ, 0DTG4ZZ, 0DTGFZZ, 0DTH0ZZ, 0DTH4ZZ, 0DTK0ZZ, 0DTK4ZZ, 0DTL0ZZ, 0DTL4ZZ, 0DTLFZZ, 0DTM0ZZ, 0DTM4ZZ, 0DTMFZZ, 0DTN0ZZ, 0DTN4ZZ, 0DTNFZZ |
| Ileostomy or Colostomy | 0D18074, 0D180J4, 0D180K4, 0D180Z4, 0D18474, 0D184J4, 0D184K4, 0D184Z4, 0D18874, 0D188J4, 0D188K4, 0D188Z4, 0D19074, 0D190J4, 0D190K4, 0D190Z4, 0D193J4, 0D19474, 0D194J4, 0D194K4, 0D194Z4, 0D19874, 0D198J4, 0D198K4, 0D198Z4, 0D1A074, 0D1A0J4, 0D1A0K4, 0D1A0Z4, 0D1A3J4, 0D1A474, 0D1A4J4, 0D1A4K4, 0D1A4Z4, 0D1A874, 0D1A8J4, 0D1A8K4, 0D1A8Z4, 0D1B074, 0D1B0J4, 0D1B0K4, 0D1B0Z4, 0D1B3J4, 0D1B474, 0D1B4J4, 0D1B4K4, 0D1B4Z4, 0D1B874, 0D1B8J4, 0D1B8K4, 0D1B8Z4, 0D1E074, 0D1E0J4, 0D1E0K4, 0D1E0Z4, 0D1E474, 0D1E4J4, 0D1E4K4, 0D1E4Z4, 0D1E874, 0D1E8J4, 0D1E8K4, 0D1E8Z4, 0D1H074, 0D1H0J4, 0D1H0K4, 0D1H0Z4, 0D1H3J4, 0D1H474, 0D1H4J4, 0D1H4K4, 0D1H4Z4, 0D1H874, 0D1H8J4, 0D1H8K4, 0D1H8Z4, 0D1K074, 0D1K0J4, 0D1K0K4, 0D1K0Z4, 0D1K3J4, 0D1K474, 0D1K4J4, 0D1K4K4, 0D1K4Z4, 0D1K874, 0D1K8J4, 0D1K8K4, 0D1K8Z4, 0D1L074, 0D1L0J4, 0D1L0K4, 0D1L0Z4, 0D1L3J4, 0D1L474, 0D1L4J4, 0D1L4K4, 0D1L4Z4, 0D1L874, 0D1L8J4, 0D1L8K4, 0D1L8Z4, 0D1M074, 0D1M0J4, 0D1M0K4, 0D1M0Z4, 0D1M3J4, 0D1M474, 0D1M4J4, 0D1M4K4, 0D1M4Z4, 0D1M874, 0D1M8J4, 0D1M8K4, 0D1M8Z4, 0D1N074, 0D1N0J4, 0D1N0K4, 0D1N0Z4, 0D1N3J4, 0D1N474, 0D1N4J4, 0D1N4K4, 0D1N4Z4, 0D1N874, 0D1N8J4, 0D1N8K4, 0D1N8Z4 |
| Diabetes Mellitus | E1010, E1011, E10630, E10638, E10641, E10649, E1065, E1069, E108, E109, E1100, E1101, E1110, E1111, E11630, E11638, E11641, E11649, E1165, E1169, E118, E119, E1300, E1301, E1310, E1311, E13630, E13638, E13641, E13649, E1365, E1369, E138, E139, E1021, E1022, E1029, E10311, E10319, E10321, E103211, E103212, E103213, E103219, E10329, E103291, E103292, E103293, E103299, E10331, E103311, E103312, E103313, E103319, E10339, E103391, E103392, E103393, E103399, E10341, E103411, E103412, E103413, E103419, E10349, E103491, E103492, E103493, E103499, E10351, E103511, E103512, E103513, E103519, E103521, E103522, E103523, E103529, E103531, E103532, E103533, E103539, E103541, E103542, E103543, E103549, E103551, E103552, E103553, E103559, E10359, E103591, E103592, E103593, E103599, E1036, E1037X1, E1037X2, E1037X3, E1037X9, E1039, E1040, E1041, E1042, E1043, E1044, E1049, E1051, E1052, E1059, E10610, E10618, E10620, E10621, E10622, E10628, E1121, E1122, E1129, E11311, E11319, E11321, E113211, E113212, E113213, E113219, E11329, E113291, E113292, E113293, E113299, E11331, E113311, E113312, E113313, E113319, E11339, E113391, E113392, E113393, E113399, E11341, E113411, E113412, E113413, E113419, E11349, E113491, E113492, E113493, E113499, E11351, E113511, E113512, E113513, E113519, E113521, E113522, E113523, E113529, E113531, E113532, E113533, E113539, E113541, E113542, E113543, E113549, E113551, E113552, E113553, E113559, E11359, E113591, E113592, E113593, E113599, E1136, E1137X1, E1137X2, E1137X3, E1137X9, E1139, E1140, E1141, E1142, E1143, E1144, E1149, E1151, E1152, E1159, E11610, E11618, E11620, E11621, E11622, E11628, E1321, E1322, E1329, E13311, E13319, E133211, E133212, E133213, E133219, E133291, E133292, E133293, E133299, E133311, E133312, E133313, E133319, E133391, E133392, E133393, E133399, E133411, E133412, E133413, E133419, E133491, E133492, E133493, E133499, E133511, E133512, E133513, E133519, E133521, E133522, E133523, E133529, E133531, E133532, E133533, E133539, E133541, E133542, E133543, E133549, E133551, E133552, E133553, E133559, E133591, E133592, E133593, E133599, E1336, E1337X1, E1337X2, E1337X3, E1337X9, E1339, E1340, E1341, E1342, E1343, E1344, E1349, E1351, E1352, E1359, E13610, E13618, E13620, E13621, E13622, E13628 |
| Obesity | E6601, E6609, E661, E662, E668, E669, Z6830, Z6831, Z6832, Z6833, Z6834, Z6835, Z6836, Z6837, Z6838, Z6839, Z6841, Z6842, Z6843, Z6844, Z6845, Z6854. |
| Chronic Kidney Disease | N181, N182, N183, N1830, N1831, N1832, N184, N185, N186, N189, Z4901, Z4902, Z4931, Z4932, N19, N030, N031, N032, N033, N034, N035, N036, N037, N038, N039, N03A, N050, N051, N052, N053, N054, N055, N056, N057, N058, N059, N05A. |
| Nicotine Dependence | F17200, F17203, F17208, F17209, F17210, F17213, F17218, F17219, F17220, F17223, F17228, F17229, F17290, F17293, F17298, F17299, Z72, Z87891. |
| Liver Disease | I8510, I8511, I864, K7040, K7041, K7110, K7111, K7151, K7210, K7211, K7290, K7291, K765, K766, K767, K7681, B180, B181, B182, B188, B189, K700, K7010, K7011, K702, K7030, K7031, K709, K713, K714, K7150, K717, K730, K731, K732, K738, K739, K740, K741, K742, K743, K744, K745, K7460, K7469, K752, K753, K754, K7581, K7589, K759, K760, K761, K762, K763, K764, K7689, K769, K77. |
| GERD | K210, K2100, K2101, K219 |
| Chronic Pulmonary Disease | I272, I2781, I2782, I2783, I2789, I279, J40, J410, J411, J418, J42, J430, J431, J432, J438, J439, J440, J441, J449, J4520, J4521, J4522, J4530, J4531, J4532, J4540, J4541, J4542, J4550, J4551, J4552, J45901, J45902, J45909, J45990, J45991, J45998, J470, J471, J479, J60, J61, J620, J628, J630, J631, J632, J633, J634, J635, J636, J64, J65, J660, J661, J662, J668, J670, J671, J672, J673, J674, J675, J676, J677, J678, J679, J684, J701, J703. |
| Hypertension | I10, I110, I119, I120, I129, I130, I1310, I1311, I132, I150, I151, I152, I158, I159. |
| Congestive Heart Failure | I099, I110, I130, I132, I255, I420, I425, I426, I427, I428, I429, I43, I501, I5020, I5021, I5022, I5023, I5030, I5031, I5032, I5033, I5040, I5041, I5042, I5043, I50810, I50811, I50812, I50813, I50814, I5082, I5083, I5084, I5089, I509, P29 |
